# Correction: Gao et al. Effects of the NF-κB Signaling Pathway Inhibitor BAY11-7082 in the Replication of ASFV. *Viruses* 2022, *14*, 297

**DOI:** 10.3390/v17030445

**Published:** 2025-03-20

**Authors:** Qi Gao, Yunlong Yang, Yongzhi Feng, Weipeng Quan, Yizhuo Luo, Heng Wang, Jiachen Zheng, Xiongnan Chen, Zhao Huang, Xiaojun Chen, Runda Xu, Guihong Zhang, Lang Gong

**Affiliations:** 1 Key Laboratory of Zoonosis Prevention and Control of Guangdong Province, College of Veterinary Medicine, South China Agricultural University, Guangzhou 510462, China; qigao2021@scau.edu.cn (Q.G.); yunlongyang@stu.scau.edu.cn (Y.Y.); fyz@stu.scau.edu.cn (Y.F.); weipengquan@stu.scau.edu.cn (W.Q.); lawzz@stu.scau.edu.cn (Y.L.); wangheng2009@scau.edu.cn (H.W.); zhengjc@stu.scau.edu.cn (J.Z.); cxn201314@stu.scau.edu.cn (X.C.); yingwenmulu@stu.scau.edu.cn (Z.H.); xiaojunchen@stu.scau.edu.cn (X.C.); 18819255305@163.com (R.X.); 2 Research Center for African Swine Fever Prevention and Control, South China Agricultural University, Guangzhou 510642, China; 3 Maoming Branch, Guangdong Laboratory for Lingnan Modern Agriculture, Maoming 525000, China; 4 African Swine Fever Regional Laboratory of China (Guangzhou), Guangzhou 510642, China

## Error in Figure

In the original publication [1], there was a mistake in Figure 7 (Effect of BAY11-7082 on p-IκB protein after ASFV infection) as published.

Upon review, we identified the incorrect placement of last row of Western Blotting results in Figure 7 of the article. We kindly request that the incorrect images be replaced with the corrected versions. The correction this time only replaced Figure 7, which does not affect the publication’s conclusion; thus, there is no need to modify the original text.

The corrected Figure 7 (Effect of BAY11-7082 on p-IκB protein after ASFV infection) is seen below. The authors state that the scientific conclusions are unaffected. This correction was approved by the Academic Editor. The original publication has also been updated.

## Figures and Tables

**Figure 7:**
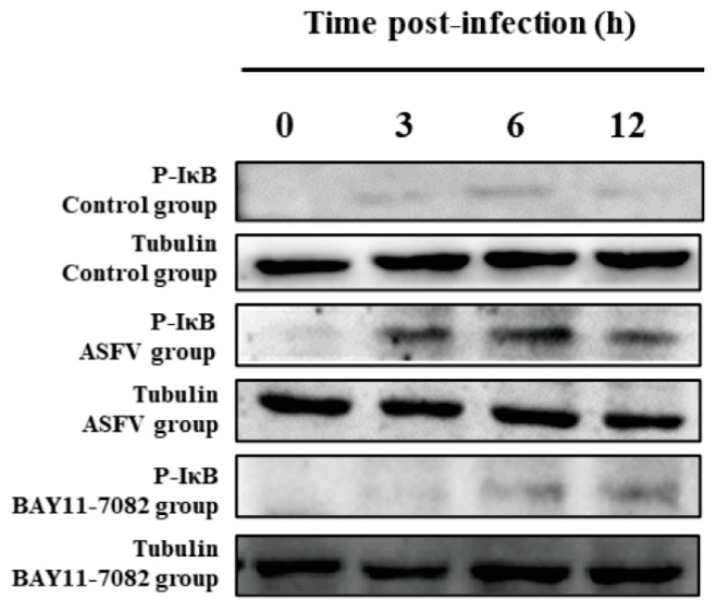
Effect of BAY11-7082 on p-IκB protein after ASFV infection. Western blotting was used to measure the expression of the p-IκB protein in the cytoplasm at 3, 6, and 12 h in each group of PAMs. Levels of p-IκB increased after ASFV infection relative to controls, and this effect was abrogated by the NF-κB inhibitor BAY11-7082. Tubulin expression was used as a positive control.
