# Supplementary material for: Effects of the NF-κB Signaling Pathway Inhibitor BAY11-7082 in the Replication of ASFV
Source: Viruses. 2022 Jan 31;14(2):297. doi: 10.3390/v14020297 (PMC8877168; doi:10.3390/v14020297)
Supplement: Supplementary file 1 [file viruses-14-00297-s001.zip › viruses-1534315-supplementary.pdf]

Supplementary Materials

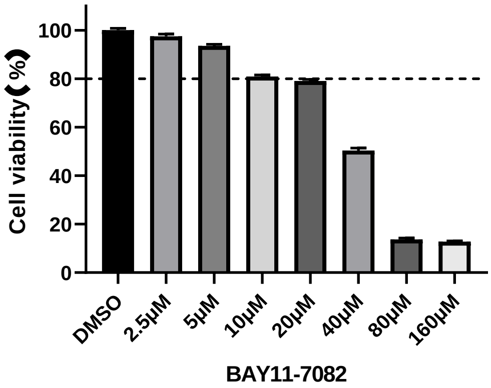

Supplementary Figure S1. Detection of cell viability of inhibitor BAY11-7082.

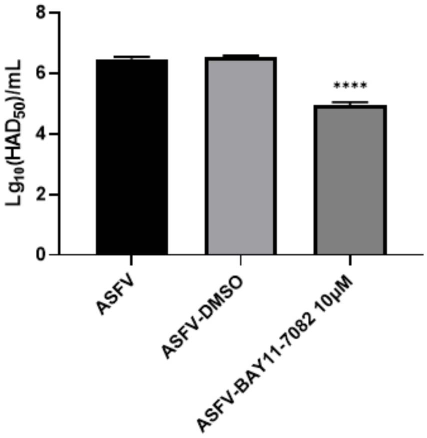

Supplementary Figure S2. Detection of virus titers. Changes in ASFV virus titers before and after inhibitor BAY11-7082 treatment. Each datum represents results of three independent experiments (means ± SD). The images above represent three independent IFA trials with similar results. Significant differences com-pared with the control group are denoted by **** ($p < 0.001$).
